# Growth Performance, Serum Biochemical Profile, Oxidative Status, and Fertility Traits in Male Japanese Quail Fed on Ginger (*Zingiber officinale*, Roscoe) Essential Oil

**DOI:** 10.1155/2018/7682060

**Published:** 2018-06-28

**Authors:** Tchoffo Herve, Kana Jean Raphaël, Ngoula Ferdinand, Folack Tiwa Laurine Vitrice, Adoum Gaye, Moussa Mahamat Outman, Ngouozeu Moyo Willy Marvel

**Affiliations:** ^1^Animal Physiology and Health Research Unit, Faculty of Agronomy and Agricultural Sciences, University of Dschang, P.O. Box 188, Dschang, Cameroon; ^2^Animal Nutrition and Production Research Unit, Faculty of Agronomy and Agricultural Sciences, University of Dschang, P.O. Box 188, Dschang, Cameroon

## Abstract

This study was designed to evaluate the effect of ginger (*Zingiber officinale*, Rosc.) essential oil on growth performance, serum biochemical profile, oxidative stress, and histological structure of testes and fertility traits in Japanese quail (*Coturnix coturnix japonica*). 96 three-week-old male Japanese quail weighing between 120 and 130 g were randomly assigned to 4 dietary treatment groups in a completely randomized design. Each group was divided into 4 replicates of 6 quails. Quails in control group received orally 100 *μ*l/kg bw of distilled water, while the three test groups received, respectively, by gastric intubation 50, 100, and 150 *μ*l/kg bw of ginger essential oil. At 12 weeks old, twelve birds per treatment were randomly selected and fasted for 24 hours, weighed, and slaughtered to assess organ and biochemical parameters. At the same period, 4 mature male quails per treatment were chosen at random and individually housed in cages, each with four untreated females for fertility and hatchability traits. The main results revealed that growth characteristics were not markedly (*P* > 0.05) affected by essential oil whatever the dose. The left testis weight increased significantly (*P* < 0.05) with 100 and 150 *μ*l/kg bw of essential oil compared to the control. The serum content in total cholesterol and triglycerides, the liver weight, the serum content in transaminases, and malondialdehyde decreased in treated quails. The serum content in total protein and globulin and the antioxidant enzymes activities increased in treated birds compared to the control. The histological changes in the testis were less visible in treated Japanese quails. At the doses of 100 and 150 *μ*l/kg bw, this essential oil induced a significant increase (*P* < 0.05) in fertility rate compared to the control. Under the conditions of this study, the ginger rhizomes essential oil can be used in poultry to reduce the lipid peroxidation in reproductive tissues and improve the fertility traits.

## 1. Introduction

The lack of the intestinal reservoir containing microorganisms that can inhibit the growth of pathogenic microorganisms and degrading toxins before their intestinal absorption in birds [1] led to the massive use of antibiotics in the poultry industry to improve growth performances, as well as reducing morbidity and mortality. Since 2006, the European Union and the authorities of many countries in the world banded the use of antibiotics as feed additives in livestock because of speculated risk in generating antibiotic resistance in pathogenic microbiota [2]. As a result, studies on natural products such as essential oils produced by aromatic plants, due to their diverse biological activities, have recently gained a great attention [3–5]. Among those aromatic plants,* Z. officinale* is used worldwide as spice and medicinal plant. The* Z. officinale* phytoconstituents essentially made up by flavonoid, phenolic acid, and terpenoid possesses many important pharmacological activities such as cardioprotective, anti-inflammatory, antimicrobial, antioxidant, antiproliferative, immunomodulatory, neuroprotective, and hepatoprotective properties [6] which can be used in animal farm to boost growth, reduce oxidative stress and histological alteration in vital organs, and subsequently improve fertility. In poultry, producing the maximum number of fertile eggs and viable chicks is a major concern. The health of testes plays a central role in sperm fertilizing capability which is positively correlated to hatchability rate. Testicular weight is highly correlated to the physiological reproductive status in poultry breeders [7]. A direct relationship has been established between sperm production and testes weight in boiler breeders [8]. Ferrouk et al. [9] reported that testicular size is the primary endpoint for spermatogenesis since seminiferous tubules and germinal elements take about 98% of the total testis mass. The beneficial effects of ginger powder in poultry growth performances, egg production and quality, carcass traits, and blood biochemistry parameters can be attributed to the phenolics and flavonoids compounds present [5, 10]. However, up to now few studies have been designed to assess the efficiency of these actives compounds on poultry reproduction.

The objective of this study was to find out the effects of graded levels of ginger roots essential oil on growth and reproductive performances in male Japanese quail.

## 2. Materials and Methods

### 2.1. Study Area

This study was carried out at the poultry unit in the Teaching and Research Farm of the University of Dschang, Cameroon. This farm is located at 5°26′ north and 10°26′ EST and at an altitude of 1420 m above sea level. Annual temperature varies between 10°C and 25°C. Rainfall ranges from 1500 to 2000 mm per annum over a 9-month rainy season (March to November).

### 2.2. Origin of Essential Oil

Fresh ginger rhizomes were harvested from Santchou (LN 5°16′55′′, LE 9°58′27′′) in the Menoua division, West Region of Cameroon. Oil extraction was done by hydrodistillation in PYTORICA Laboratory, Bonapriso, Douala Cameroon, as described by Wang and Weller [11].

### 2.3. Phytochemical Screening of Essential Oil

The phytochemical screening of essential oil (Table 1) was done as described by Banso and Ngbede [12] and Ngbede et al. [13].

### 2.4. Animals and Experimental Design

Ninety-six 3-week-old male Japanese quail* (Coturnix coturnix japonica)* weighing between 120 and 130 g, produced from a parent stock in the Teaching and Research Farm of the University of Dschang, were used for testing. Each quail was identified by a ring bearing his number in one of its paws.

At the beginning of the experiment, quails were weighed and randomly assigned to 4 dietary treatment groups in a completely randomized design. Each group was divided into 4 subgroups of 6 quails. Quails in control group received orally 100 *μ*l/kg bw of distilled water, while birds in the other three test groups, during the same period, respectively, received by gastric intubation 50, 100, and 150 *μ*l/kg body weight of essential oil. At 12 weeks old, twelve birds per treatment were randomly selected and slaughtered for organs characteristics assessment and blood samples were collected for biochemical analysis. At the same period, 4 male quails per treatment with hypertrophy of cloacal gland were selected and individually housed in the same cage with four untreated females and reared under the same conditions, for fertility, hatchability traits, and chick weight assessment. During the experimental period, feed (Table 2) and water were offered ad libitum to quail in adapted equipment.

This study was carried out in strict accordance with recommendations of institutional guidelines for the care and use of laboratory animals. Quails were humanly handled with respect to the ethical standards laid down in the 1964 Declaration of Helsinki and its later amendments.

### 2.5. Growth Characteristics

Feed intake and life body weight for individual quail were recorded weekly; body weight gain was obtained by the difference in life body weight of two consecutive weeks according to the procedures of McDonald et al. [14]. Feed conversion ratio was obtained by dividing weekly feed intake by weekly body weight gain.

### 2.6. Blood Sampling and Organ Weights

At the end of the experiment, 12 quails per treatment were randomly selected and fasted for 24 hours, weighed, and slaughtered as described by Jourdain [15]. Blood samples were collected from the jugular vein in nonheparinized tubes; the serum isolated was stored at −20°C for biochemical analysis.

Testes and liver of slaughtered quails were carefully removed, rid of adipose tissue, blotted dry, and weighed separately using a scale of 160 g capacity and 10^−3 ^g precision. The relative organ weight was calculated as follows:

(1) Relative organ weight %=Organ weight (mg)Live body weight g×100.

The left testis was homogenized in a known volume of cold 0.9% NaCl followed by a centrifugation (3000 rpm, 30 min), and the resultant supernatants were subsequently stored at −20°C for antioxidant status assessment.

### 2.7. Biochemical Analysis

Serum content in proteins was determined by Biuret method [16]. Alanine aminotransferase (ALT) and aspartate aminotransferase (AST) contents were evaluated by the enzymatic analysis method using commercial kits CHRONOLAB, Ref. 101-0255 and CHRONOLAB, Ref. 101-0256, respectively. Total cholesterol and triglycerides were determined by colorimetric methods using commercial kits CHRONOLAB, Ref. 101-0576 and CHRONOLAB, Ref. 101-0241, respectively. Total globulins were calculated as described by Abdel-Fattah et al. [17].

### 2.8. Oxidative Stress Characteristics

The malondialdehyde concentration was evaluated by the thiobarbituric acid method [18]. Superoxide dismutase (SOD), catalase (CAT), and reduced glutathione (GSH) activities were evaluated as described by Misra and Fridovich [19], Sinha [20], and Ellman and Fiches [21], respectively.

### 2.9. Histology

Histological screening of testes was carried out as described by Wolfang [22] for organ or tissues. Briefly, the right testis was fixed by immersion in Bouin solution for 1 week and then washed, dehydrated in ascending grade alcohol bath, clarified in xylene immersion, and embedded in paraffin. Sections of 5 *μ*m were stained with hematoxylin-eosin for histological observations under a light microscope (400x).

### 2.10. Fertility and Hatchability Traits

A total of 56 eggs per group were collected during 8 days, weighted individually, and incubated. After artificial incubation for 19 days, all unhatched eggs were cracked and classified as infertile or embryonic mortality. The fertility rate was then calculated by dividing the number of fertile eggs over the total number of eggs incubated. The individual chick's weight was obtained by dividing the total chick's weight over the number of chicks hatch per group.

### 2.11. Statistical Analysis

The statistical analysis was carried out using the SPSS 20.0 software. Results were expressed as mean ± standard deviation. Differences between groups were assessed using one-way ANOVA followed by Duncan post hoc test with the significance level set at 0.05. *P* value was done using the Student's *t*-test. A *P* value of less than 0.05 was considered as significant. The normality of data was tested by the Shapiro-Wilk Test and the relationships between different parameters were highlighted by the correlation coefficient of Bravais-Pearson.

## 3. Results

### 3.1. Growth Characteristics

As shown in Table 3, feed intake, live body weight, body weight gain, and feed conversion ratio (FCR) were not significantly affected (*P* > 0.05) by the doses of essential oil used. However, the values of these growth characteristics tend to decrease in treated quails compared to the quails in control group.

### 3.2. Organs Weight

The relative weight of the left testis increased significantly (*P* < 0.05) in quails treated with essential oil at 100 and 150 *μ*l/kg bw compared to quails in the control group and quails fed on the smallest dose of essential oil (50 *μ*l/kg bw). The relative weight of the right testis in treated quails was comparable (*P* > 0.05) to that of the quails in the control group, although this weight tended to increase in birds treated with 100 and 150 *μ*l/kg of bw (Table 3).

The relative weight of the liver recorded in quails treated with oil whatever the dose was comparable (*P* > 0.05) to that of the quails in the control group. However, this weight tends to decrease with 100 and 150 *μ*l/kg bw compared to the weight of liver recorded in the control group and quails fed on the smallest dose of the essential oil (50 *μ*l/kg of bw) (Table 3).

### 3.3. Serum Biochemical Parameters

The ginger essential oil whatever the dose induced a significant increase (*P* < 0.05) in protein and globulin serum contents compared to the control. However, the serum contents in protein and globulin recorded with 100 and 150 *μ*l/kg bw were comparable and significantly higher (*P* < 0.05) relative to the quantity recorded in quails treated with the lowest dose of this essential oil (50 *μ*l/kg bw) (Table 4). The alanine aminotransferase (ALT) activity decreased significantly (*P* < 0.05) in quails treated with essential oil whatever the dose compared to the control group. Although statistically comparable, the activity of ALT tends to decrease in quails treated with 150 *μ*l/kg bw as compared to 50 and 100 *μ*l/kg bw. The oral administration of ginger essential oil induced a decrease in aspartate aminotransferase (AST) activity relative to the control. However, this decrease was significant only with 150 *μ*l/kg bw compared to other essential oil doses. The serum content in total cholesterol decreased significantly (*P* < 0.05) and linearly with the increasing doses of the essential oil relative to the control. Essential oil significantly (*P* < 0.05) decreases triglyceride content compared to the control (Table 4).

### 3.4. Antioxidant Status

The malondialdehyde (MDA) level in the treated quails decreased significantly (*P* < 0.05) and linearly with the increasing doses of the essential oil compared to the control group. The oral administration of the essential oil induced an increase in superoxide dismutase (SOD) activity relative to the control. However, this increase was significant only with 150 *μ*l/kg bw compared to all other doses (Table 4). The glutathione (GSH) activities in quails treated with essential oil at 100 and 150 *μ*l/kg bw were comparable and significantly (*P* < 0.05) higher than activities recorded in control group and the group of quails treated with 50 *μ*l/kg bw. The catalase (CAT) activities were comparable with 100 and 150 *μ*l/kg bw but significantly higher (*P* < 0.05) than CAT activity recorded in the control group (Table 4).

### 3.5. Histological Structure of the Testes

**Figure 1:**
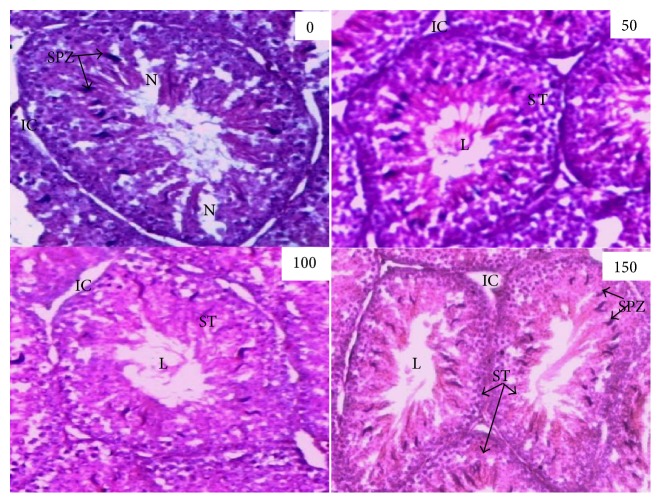
Histological structure of Japanese quail testes as affected by ginger roots essential oil (400x). 0 (control); 50, 100, and 150: doses of ginger roots essential oil (*μ*l/kg body weight). SPZ: spermatozoa; ST: seminiferous tubules; IC: interstitial cells; L: lumen; and N: necrosis.

The testes of the quails in the control group showed slight alterations of the seminiferous tubules with necrosis. The oral administration of the essential oil whatever the dose improved the histological structure of the testes compared to the control. The histological changes observed in the testes of the treated quails whatever the dose were almost identical (Figure 1).

### 3.6. Fertility and Hatchability Traits

The fertility rate of quails treated with the essential oil except the lowest dose (50 *μ*l/kg bw) significantly increased (*P* < 0.05) compared to the control group. However, the fertility rate of quails treated with 100 *μ*l/kg bw is comparable to the fertility of quails treated with the highest dose of essential oil (150 *μ*l/kg bw). The relative weight of the testes is positively and significantly correlated to the fertility rate (*ρ* = +0.99, *P* < 0.01); the same observation was made between GSH and the fertility rate (*ρ* = +0.95, *P* < 0.05) (Table 5).

The oral administration of ginger rhizome essential oil at the entire used dose tends to increase the hatching rate of fertile eggs and the total hatch rate compared to the control (Table 6). However, the highest fertile egg hatched and total hatch rates were recorded in quails treated with 100 *μ*l/kg bw. A positive and nonsignificant correlation was recorded between the fertility rate and the total hatch rate (*ρ* = +0.65; *P* > 0.05) (Table 5).

The embryonic mortality rate was not significantly affected (*P* > 0.05) by the doses of the essential oil used. However, it tends to decrease with essential oil whatever the dose compared to the control. The lowest embryonic mortality rate was recorded in quails treated with 100 *μ*l/kg bw. The ginger essential oil whatever the dose induced a nonsignificant (*P* > 0.05) increase in chick's weight compared to the control. However, the highest chick weight was recorded with 100 *μ*l/kg bw of the essential oil (Table 6).

## 4. Discussion

The present study revealed that feed intake, live body weight, and body weight gain were not significantly affected by the ginger essential oil treatments over the 12-week period. This observation agreed with the findings of Dieumou et al. [23] who recorded no significant differences in feed intake, body weight gain, and feed conversion ratio with 10, 20, and 40 mg/kg bw of ginger essential oil for seven consecutive weeks in broilers. The findings of Fakhim et al. [5] also revealed no improvement in body weight gain of chickens fed on ginger supplements compared to the control group. Feed conversion ratio decreased nonsignificantly and linearly with the increasing doses of essential oil compared to the control. This effect could be explained by the various properties of phenolic and flavonoid compounds of the ginger essential oil in the digestive system of the animal. These bioactive compounds possess antimicrobial and antioxidant properties that allow them to reduce the free radical and pathogenic microbe's attacks and a better use of the nutrients in the digestive tract.

The present study revealed that the oral administration of essential oil whatever the dose increased significantly the serum content in total protein and globulin compared to the control. This result is consistent with the findings of Zhang et al. [24] with 5 g/kg of ginger rhizomes powder in broiler feed. The present results contradicted the findings of many other studies which recorded a significant decrease in serum protein and globulin levels in chickens fed on diet supplemented with ginger rhizomes powder [25, 26]. The differences between these studies could be the result of difference in the doses used, the shape of the plant (powder, essential oil, etc.), the route of administration, and the experimental conditions. The increase in total protein and globulin may be due to phenolic components, including gingerol, shogaols, gingerdiol, gingerdione, and some related phenolic ketone derivatives of ginger essential oil [4], which have powerful antioxidant and immunostimulatory properties that allow them to improve immune responses.

Serum content in aspartate aminotransferase (AST) and alanine aminotransferase (ALT) significantly decreased in quails fed on ginger rhizomes essential whatever the dose compared to the control birds. These results are consistent with the findings of Malekizadeh et al. [27] who reported a significant decrease in serum AST and ALT levels in Hyline leghorns (W-36) fed on diet supplemented with 3% ginger rhizome powder for 9 weeks. The decrease in the transaminases levels suggested that the doses of oil administered were not toxic and regulated the liver activity of the quails. According to Zounongo [28], an increase in serum transaminase levels indicates hepatic cytolysis. In fact, the weight of the liver in the present study tends to decrease in treated quails at the doses of 100 and 150 *μ*l/kg bw.

The malondialdehyde (MDA) as an indicator of lipid peroxidation and oxidative stress significantly decreased in all treatments groups compared to the control. The activities of the antioxidant enzymes (superoxide dismutase, catalase, and reduced glutathione) increased with the oral administration of this essential oil in quails. Antioxidant activity is one of the main defense systems of the body against the harmful effects of reactive oxygen species in animals. The antioxidant action of ginger has been attributed to the protective actions of ginger bioactive substances against free radical attack [29, 30]. This antioxidant action of ginger essential oil subsequently reduced the lipid peroxidation responsible for apoptosis in spermatogenic cells. Indeed, the testicular histology in quails treated with ginger rhizomes essential oil revealed structural improvements compared to the control birds that showed mild necrosis. In accordance with this result, Zancan et al. [31] revealed that ginger has protective effects against cisplatin-induced testicular damage and oxidative stress in animals. Shanoon [32] mentioned that the ginger rhizomes bioactive molecules improve testicular structure by increasing the thickness of the seminiferous tubules and the germ cell membrane and consequently increase testicular weight, viability, and motility of the spermatozoa.

All the above-mentioned properties contribute to the fertility of the quail treated with different doses of the ginger roots essential oil, resulting in an increase in the fertility rate. The present results are consistent with the findings of Ezzat et al. [33] in Cobb 500 parent broiler treated with ginger rhizome powder at the doses of 2.5 and 5 g/kg of feed for 14 weeks. This elevating effect can be attributed to the ginger essential oil active compounds (alkaloids and terpenes) which improve the characteristics of the spermatozoa and, consequently, make them more active in the female genital tract. According to Froman et al. [34], a higher proportion of highly motile spermatozoa enter the sperm storage tubules of female birds, resulting in a high proportion of fertile eggs. The ginger rhizomes essential oil whatever the dose increased the hatching rate of fertile eggs. This effect could be related to sperm characteristics (membrane and DNA integrity, viability, and mobility) enhanced by the phenolic compounds of the essential oil. Indeed, the sperm cell membrane is particularly rich in polyunsaturated fatty acid which predisposes them to lipid peroxidation by reactive oxygen species, which is associated with male infertility [35]. Large amounts of radical oxygen species have been shown to interfere with the integrity of sperm DNA and thereby influence embryo development [36]. The supplementation of the male bird diet with ginger rhizomes essential oil rich in antioxidant property could reduce the impairment in sperm membrane and DNA. In accordance with the present result, Hoe et al. [37] mentioned that the ginger essential oil protects the DNA from oxidation by hydrogen peroxide and from the harmful effects of the reactive oxygen species. This ginger essential oil activity consequently increases the fertility and hatching rate and reduces the mortality rate. The present results revealed a positive correlation between the fertility and the hatching rate of fertile eggs suggesting an improvement in this parameter with the increasing rate of the fertility.

## 5. Conclusion

The present results revealed that ginger rhizome essential oil can be used in quails to reduce the lipid peroxidation in reproductive cells and promote fertility without adverse effects on growth performances.

## Figures and Tables

**Table 1:** Phytochemical constituents of ginger essential oil.

Constituents	(+) present; (−) absent
Alkaloids	+
Triterpenoid	+
Steroid	−
Flavonoid	+
Phenol	+

**Table 2:** Composition of the experimental diet.

Ingredients	Amount (kg/100 kg)
Maize	60
Wheat bran	4.5
Soybean meal	22
Fishmeal	4.5
Oeister shell	2
Bone meal	2
Premix 5%^*∗*^	5
Total	100

Calculated chemical composition	

Crude protein (%)	20.15
Metabolizable energy (Kcal/Kg)	2906.80
Calcium (%)	2.03
Phosphorus (%)	1.27
Lysine (%)	0.44
Methionine (%)	0.14
Sodium (%)	0.22

^*∗*^Premix 5%: mixture of vitamins A, B complex, D, K, and E.

**Table 3:** Effects of graded level of ginger roots essential oil on growth characteristics and relative organ weightsof male Japanese quail.

Parameters	Essential oil doses (*μ*l/kg body weight)				
	Control (*n* = 12)	50 (*n* = 12)	100 (*n* = 12)	150 (*n* = 12)	*P* value
*Growth characteristics *					
Feed intake (g)	907 ± 43.54	910.13 ± 48.19	857.63 ± 47.83	889.15 ± 37.75	0.17
Live body weight (g)	209.27 ±15.02	207.45 ± 24.90	204.27 ± 15.27	202.25 ± 17.72	0.80
Body weight gain (g)	225.29 ± 46.75	227.86 ± 37.98	219.43 ± 46.59	233.14 ± 45.47	0.95
FCR	5.98 ± 2.07	5.79 ± 1.79	5.11 ± 0.69	4.63 ± 0.89	0.39
*Organ weights (g/100 g bw)*					
Liver	1.55 ± 0.33	1.57 ± 0.29	1.46 ± 0.39	1.47 ± 0.36	0.85
Pair of testes	2.01 ± 0.48^a^	2.08 ± 0.16^a^	2.63 ± 0.21^b^	2.54 ± 0.32^b^	0.01
Right testis	1.00 ± 0.24	1.04 ± 0.16	1.18 ± 0.23	1.14 ± 0.22	0.56
Left testis	1.01 ± 0.26^a^	1.05 ± 0.17^a^	1.46 ± 0.07^b^	1.41 ± 0.33^b^	0.01

^a,b^On the same line, means with the same letter were not significantly different (*P* > 0.05). *n* = number ofquails; FCR = feed conversion ratio.

**Table 4:** Effects of graded levels of ginger essential oil on serum biochemical and testes oxidative stress characteristics

Parameters	Essential oil doses (*μ*l/kg body weight)				
	Control (*n* = 12)	50 (*n* = 12)	100 (*n* = 12)	150 (*n* = 12)	*P* value
*Serum metabolites*					
Serum proteins (g/dl)	2.06 ± 0.04^a^	2.38 ± 0.19^b^	2.66 ± 0.19^c^	2.70 ± 0.35^c^	0.00
Globulins (g/dl)	0.16 ± 0.05^a^	0.31 ± 0.06^b^	0.49 ± 0.07^c^	0.48 ± 0.16^c^	0.00
AST (U/L)	168.00 ± 20.24^b^	146.24 ± 15.11^ab^	163.24 ± 11.29^ab^	137.49 ± 16.88^a^	0.00
ALT (U/L)	57.50 ± 10.49^b^	42.85 ± 3.46^a^	40.00 ± 10.14^a^	33.43 ± 6.07^a^	0.00
Total cholesterol (mg/dl)	127.27 ± 7.98^d^	118.09 ± 5.41^c^	110.06 ± 5.83^b^	100.27 ± 1.98^a^	0.00
Triglycerides (mg/dl)	73.46 ± 10.39^b^	57.18 ± 4.43^a^	56.91 ± 6.49^a^	56.07 ± 6.16^a^	0.00
*Testes oxidative stress (gram of tissue)*					
MDA	97.79 ± 9.60^c^	82.20 ± 9.93^b^	52.49 ± 8.39^a^	50.57 ± 17.83^a^	0.00
SOD	2.92 ± 0.48^a^	3.47 ± 0.33^a^	3.44 ± 0.55^a^	4.08 ± 0.23^b^	0.00
GSH	183.73 ± 35.15^a^	201.47 ± 17.74^a^	265.07 ± 48.25^b^	274.02 ± 54.16^b^	0.00
CAT	2.82 ± 0.31^a^	3.36 ± 0.73^ab^	3.78 ± 0.63^b^	3.60 ± 0.89^b^	0.04

^a,b,c,d^On the same line, means with the same letter were not significantly different (*P* > 0.05); *n* = number ofquails; MDA = malondialdehyde; GSH = reduced glutathione; CAT = catalase; SOD = superoxide dismutase; AST = aspartate aminotransferase; ALT = alanine aminotransferase; values are presented as means ± standard deviation.

**Table 5:** Correlations between testes weight, GSH, sperm motility, total hatch, and fertility rate.

Parameters	Pair of testes relative weight	Fertility rate	Total hatch rate
Fertility rate	0.99^*∗∗*^	-	0.65
Sperm motility	0.99^*∗*^	0.99^*∗∗*^	-
GSH	0.98^*∗*^	0.95^*∗*^	-

^*∗*^The correlation is significant at the 0.05 level. ^*∗∗*^The correlation is significant at the 0.01 level.

**Table 6:** Effects of graded levels of ginger essential oil on fertility rate and hatchability traits in Japanese quail.

Parameters	Essential oil doses (*μ*l/kg body weight)				
	Control (*n* = 12)	50 (*n* = 12)	100 (*n* = 12)	150 (*n* = 12)	*P* value
Fertility (%)	81.89 ± 4.91^a^	82.14 ± 3.11^a^	90.71 ± 4.90^b^	88.54 ± 2.02^b^	0.01
Hatchability of fertile eggs (%)	65.00 ± 14.01	78.93 ± 6.53	84.29 ± 11.19	72.50 ± 15.09	0.18
Total hatchability (%)	60.71 ± 21.43	71.43 ± 10.16	78.57 ± 27.36	67.14 ± 14.47	0.63
Embryonic mortality (%)	10.08 ± 2.23	9.48 ± 3.72	9.46 ± 1.19	9.66 ± 0.59	0.99
Chick's weight (g)	8.08 ± 1.04	8.17 ± 1.07	8.42 ± 1.22	8.33 ± 0,76	0.40

^a,b^On the same line, means with the same letter were not significantly different (*P* > 0.05). *n* = number ofquails.

## Data Availability

The data sets used during the current study are available from the corresponding author on reasonable request.
